# Optimizing an Acceptance and Commitment Therapy Microintervention Via a Mobile App With Two Cohorts: Protocol for Micro-Randomized Trials

**DOI:** 10.2196/17086

**Published:** 2020-09-23

**Authors:** Emily B Kroska, Sydney Hoel, Amanda Victory, Susan A Murphy, Melvin G McInnis, Zachary N Stowe, Amy Cochran

**Affiliations:** 1 Department of Psychological and Brain Sciences University of Iowa Iowa City, IA United States; 2 Department of Psychiatry University of Wisconsin–Madison Madison, WI United States; 3 Department of Psychiatry University of Michigan–Ann Arbor Ann Arbor, MI United States; 4 Department of Statistics Harvard University Cambridge, MA United States; 5 Department of Population Health Sciences University of Wisconsin–Madison Madison, WI United States; 6 Department of Math University of Wisconsin–Madison Madison, WI United States

**Keywords:** acceptance and commitment therapy, clinical trial, mobile apps, bipolar disorder, students, mobile phone

## Abstract

**Background:**

Given gaps in the treatment of mental health, brief adaptive interventions have become a public health imperative. Transdiagnostic interventions may be particularly appropriate given high rates of medical comorbidity and the broader reach of transdiagnostic therapies. One such approach utilized herein is acceptance and commitment therapy (ACT), which is focused on increasing engagement with values, awareness, and openness to internal experiences. ACT theory posits that experiential avoidance is at the center of human suffering, regardless of diagnosis, and, as such, seeks to reduce unworkable experiential avoidance.

**Objective:**

Our objective is to provide the rationale and protocol for examining the safety, feasibility, and effectiveness of optimizing an ACT-based intervention via a mobile app among two disparate samples, which differ in sociodemographic characteristics and symptom profiles.

**Methods:**

Twice each day, participants are prompted via a mobile app to complete assessments of mood and activity and are then randomly assigned to an ACT-based intervention or not. These interventions are questions regarding engagement with values, awareness, and openness to internal experiences. Participant responses are recorded. Analyses will examine completion of assessments, change in symptoms from baseline assessment, and proximal change in mood and activity. A primary outcome of interest is proximal change in activity (eg, form and function of behavior and energy consumed by avoidance and values-based behavior) following interventions as a function of time, symptoms, and behavior, where we hypothesize that participants will focus more energy on values-based behaviors. Analyses will be conducted using a weighted and centered least squares approach. Two samples will run concurrently to assess the capacity of optimizing mobile ACT in populations that differ widely in their clinical presentation and sociodemographic characteristics: individuals with bipolar disorder (n=30) and distressed first-generation college students (n=50).

**Results:**

Recruitment began on September 10, 2019, for the bipolar sample and on October 5, 2019, for the college sample. Participation in the study began on October 18, 2019.

**Conclusions:**

This study examines an ACT-based intervention among two disparate samples. Should ACT demonstrate feasibility and preliminary effectiveness in each sample, a large randomized controlled trial applying ACT across diagnoses and demographics would be indicated. The public health implications of such an approach may be far-reaching.

**Trial Registration:**

ClinicalTrials.gov NCT04098497; https://clinicaltrials.gov/ct2/show/NCT04098497; ClinicalTrials.gov NCT04081662; https://clinicaltrials.gov/ct2/show/NCT04081662

**International Registered Report Identifier (IRRID):**

DERR1-10.2196/17086

## Introduction

### Background

Brief interventions have garnered public health attention in recent years regarding improvements in patient and provider efficiency. Many studies have indicated the effectiveness of brief interventions in creating and sustaining clinically meaningful levels of change. Several meta-analyses [1,2] and large community-based studies [3] indicate that rapid improvements in symptoms often occur after brief interventions and that change occurs at an accelerated rate when patients are provided fewer therapy sessions [4]. Wide-scale applications of effective brief approaches that reach diverse patient groups, particularly those with limited access to services, are important.

Digitally delivered interventions are promising in terms of reach, acceptability, individualization, and cost-effectiveness. Users can tailor consumption of content, seeking support when needed, and integration with the users’ context can be provided. Despite great potential and expanded capacity, many digital interventions have yet to be evaluated for effectiveness in randomized controlled trials (RCTs) [5]. Moreover, digital interventions are frequently developed for a specific mental health disorder, such as bipolar disorder [6], borderline personality disorder [7], major depressive disorder [8], anxiety disorders [9], and posttraumatic stress disorder [10]. Naturally, digital interventions that are effective across affective disorders have potential for helping a greater number of individuals. By developing a brief, digitally delivered intervention, the goal of this study is to identify whether a transdiagnostic approach could be adapted to a microintervention design, exploring the proximal impacts of interventions on mood and activity. The implications of a brief, effective, and easily disseminated mobile app are far-reaching, given large treatment gaps in mental health [11]. Furthermore, transdiagnostic interventions that target functioning over symptoms have broad potential to impact human suffering across diagnostic presentations and comorbidities. Acceptance and commitment therapy (ACT) is a transdiagnostic, process-based intervention that has established empirical support in over 300 RCTs. This manuscript presents two parallel protocols for micro-randomized trials for optimizing an ACT-based mobile app in two samples with pronounced differences in sociodemographic backgrounds and in symptom profiles: (1) individuals with bipolar disorder and (2) distressed first-generation college students.

### ACT

ACT is a transdiagnostic, mindfulness-based, and acceptance-based behavioral therapy. Its overarching goal is to increase psychological flexibility, allowing patients to behave consistently with their values, even in the presence of difficult thoughts, emotions, or other internal experiences [12]. Psychological flexibility includes awareness of internal experiences (eg, thoughts and emotions) and behaviors, openness to internal experiences, and engagement with values. Notably, the central goal of ACT is not to remove unwanted symptoms (eg, distress and depression) but to help individuals pursue a life of meaning even in the presence of such symptoms. ACT targets experiential avoidance: the inability or unwillingness to make contact with internal experiences (eg, thoughts, emotions, and memories) [12]. Avoidance provides short-term relief but exacerbates long-term experiences of the avoided stimulus in intensity and duration. Avoidance also reduces contact with valued life directions. Conversely, psychological flexibility is associated with increased well-being and reduced symptoms [13].

**Figure 1:**
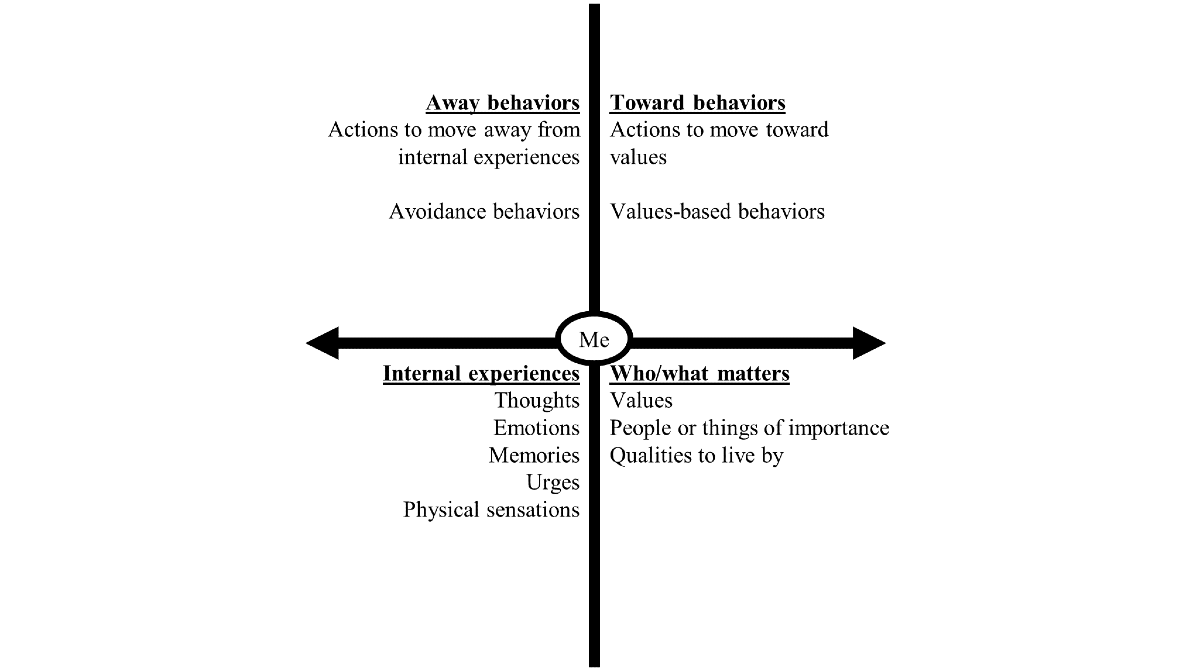
The acceptance and commitment therapy (ACT) matrix. The ACT matrix encourages awareness of one’s values, internal experiences, and the function of one’s behaviors. The top two quadrants are observable behaviors, while the bottom two quadrants are internal experiences and not observable to others. The middle circle signifies the ability to notice each of these domains, categorizing all quadrants as part of a person’s experience.

ACT has demonstrated efficacy when delivered to transdiagnostic populations and over brief periods (eg, a few weeks or digitally) [14-16]. ACT effectively treats a number of psychiatric and physical conditions, including chronic pain, depression, and anxiety [13]. For example, ACT has improved depressive symptoms in a sample of college students via an online, guided intervention [17]. An ACT-based mobile app improved psychological flexibility [18], suggesting that improvements can be achieved utilizing mobile technology [19-22]. By synthesizing processes related to ACT, the ACT matrix helps patients to identify values, values-based behaviors, internal experiences, and avoidance behaviors (see Figure 1) [23]. This tool has been utilized in brief RCTs demonstrating positive outcomes [24,25]. Despite support for ACT in brief form and via digital media, research has not yet examined microinterventions related to specific ACT processes (ie, openness, awareness, and engagement, as discussed below). Furthermore, in development of such an intervention, it is important to know whether such an approach would work *across* samples of differing backgrounds and diagnostic presentations, as would be hypothesized with a transdiagnostic approach.

### Micro-Randomized Trials

Micro-randomized trials are a new type of RCT well suited for optimizing interventions delivered via mobile apps [26,27]. In a traditional RCT, individuals are randomized once to an intervention condition. Researchers can then estimate causal effects of the intervention on outcomes. In contrast, micro-randomized trials repeatedly randomize individuals to intervention groups, and causal effects of the interventions on proximal outcomes can be estimated in a micro-randomized trial. Because randomization is repeated, the moderating role of in-the-moment information (eg, current symptoms) on causal effects of the intervention can be examined. With many mobile interventions striving to intervene *just-in-time* [28], micro-randomized trials can provide evidence on what immediate information is needed to optimize the timing of psychosocial interventions. Micro-randomized trials have evaluated physical health and substance use interventions [29-31], but they have not evaluated such interventions among bipolar individuals or distressed college students, or ones based on ACT.

### Objective

The micro-randomized trials in this study evaluate safety, feasibility, and effectiveness of optimizing an ACT-based microintervention via a mobile app over the course of 6 weeks. To investigate the possibility of optimizing mobile ACT in transdiagnostic populations, two samples with clinically distinct presentations are studied: (1) distressed first-generation college students and (2) individuals with bipolar disorder. As detailed in the Methods section below, these samples differ noticeably in the severity and chronicity of affective illness, affective symptoms manifestation, and sociodemographic characteristics. The two trials seek to examine the proximal effects of the intervention across the samples in terms of mood, perceived stress, and/or activity. The overarching goal of the trials is to determine whether optimizing an ACT-based app has potential to help with behavioral and mood changes in diverse and transdiagnostic populations. The findings bear on improving access to care, providing adjuncts to traditional psychotherapy, and increasing efficiency in delivery of psychotherapy.

## Methods

### Study Design

The two studies described in this paper share one core research design, adapted to fit each cohort. Each study uses a micro-randomized trial to evaluate safety, feasibility, and effectiveness of a mobile ACT-based intervention. This intervention consists of prompts designed to embody the central tenets of ACT. The intervention is delivered over 6 weeks through an app designed for these studies. Because the scale of delivery is small compared to a traditional intervention, we refer to this as a *microintervention.* Both studies were registered at ClinicalTrials.gov (NCT04098497 and NCT04081662).

After consenting, participants complete baseline questionnaires on symptoms, functioning, and background information. Participants are prompted to download a mobile app designed for the study. Upon opening the app, participants are provided the link and encouraged to watch a 20-minute video that introduces the core concepts of the intervention. Participants are asked to log symptoms in the app. Each time the participant logs symptoms, they have a 50% chance of receiving a microintervention, which is randomly chosen from a set of 84 questions. Participants answer questions about the activities they are currently engaged in. They are asked to identify the form of the activity (eg, walking dog) as well as the function of the behavior (ie, toward values or away from internal experiences). After the conclusion of the 6-week study, participants complete follow-up symptom scales and questionnaires.

Table 1 summarizes the study design and differences between each cohort. The difference between cohorts is the set of symptom scales used at baseline, follow-up, and in the app. For individuals with bipolar disorder, scales measure mania and depression. For distressed first-generation students, scales measure stress and depression. Sample-specific scale selection affords an opportunity to observe the relevant symptoms and to evaluate effectiveness of the microintervention in alleviating symptoms. See Table 1 [32-39] for the complete assessment battery.

**Table 1:** Summary of study design and differences between cohorts.

Design element	Bipolar cohort	College student cohort
Sample size, n	30	50
Baseline assessments (day 0)	A phone interview^a^ to complete the YMRS^b^ [34], the SIGH-D^c^ [35], and the SF-36^d^ [36]	An online assessment to complete the PSS-10^e^ [32], the PHQ-9^f^ [33], the PROMIS-29^g^ [37], the AAQ-2^h^ [38], and the CompACT^i^ [39]
In-app assessments (days 1-42)	Delivered through the app twice daily: the shortened YMRS, the shortened SIGH-D, and the ACT^j^ Activity Survey^k^	Delivered through the app twice daily: the PHQ-2^l^, the PSS-4^m^, and the ACT Activity Survey^k^
Activity tracker assessments (days 1-42)	Sleep, heart rate, and steps tracked through the Fitbit Alta HR	None
Microintervention (days 1-42)	Randomized to receive or not receive ACT microintervention^k^ after in-app assessments	Randomized to receive or not receive ACT microintervention^k^ after in-app assessments
Exit assessments (day 42)	A phone interview to complete same assessments from baseline	An online assessment to complete same assessments from baseline, along with an app engagement survey^k^
Follow-up assessments (months 3 and 6)	None	Online assessments to complete same assessments from baseline
Primary outcomes	Safety and feasibility of microintervention in terms of the following: adherence to in-app assessments (ie, feasibility); change in YMRS and SIGH-D scores from baseline to exit assessment (ie, safety)	Effectiveness, safety, and feasibility of microintervention in terms of the following: changes in responses to ACT Activity Survey as a function of whether the microintervention was delivered at a prior time point (ie, effectiveness); adherence to in-app assessments (ie, feasibility); change in proportion of individuals who meet criteria for minor or major depression on PHQ-9 from baseline to exit and from baseline to each follow-up assessment (ie, safety)
Secondary outcomes	Power for larger study based on changes in responses to ACT Activity Survey as a function of whether the microintervention was delivered at a prior time point (ie, effectiveness)	Effectiveness of microintervention in terms of changes in responses to PHQ-2 and PSS-4 scores as a function of whether the microintervention was delivered at a prior time point (ie, effectiveness)

^a^Participants are recruited from the Prechter Longitudinal Study of Bipolar Disorder and have already completed interviews to determine demographic information and health and mental illness history.

^b^YMRS: Young Mania Rating Scale.

^c^SIGH-D: Structured Interview Guide for the Hamilton Depression Rating Scale.

^d^SF-36: 36-Item Short Form Survey.

^e^PSS-10: Perceived Stress Scale 10.

^f^PHQ-9: Patient Health Questionnaire 9.

^g^PROMIS-29: Patient-Reported Outcomes Measurement Information System.

^h^AAQ2: Acceptance and Action Questionnaire-II.

^i^CompACT: Comprehensive Assessment of Acceptance and Commitment Therapy Processes.

^j^ACT: acceptance and commitment therapy.

^k^Developed for these studies.

^l^PHQ-2: Patient Health Questionnaire 2.

^m^PSS-4: Perceived Stress Scale 4.

### Randomization

Participants are repeatedly randomized to either receive an intervention or not receive an intervention throughout the study. Participants are *available* for randomization every time they complete an in-app assessment, which can be completed once in the morning and once in the evening throughout the study. Randomization occurs immediately after a participant clicks the button to submit an in-app assessment. Participants have a 50-50 chance of receiving a microintervention. Since there are a total of 84 different assessments (2 per day × 42 days), participants may be randomized to receive a microintervention for a maximum of 84 different times throughout the study. If the participant is not assigned to receive a microintervention, then they are taken to the home page. If they are assigned to receive a microintervention, then a second randomization is performed to determine which of the 84 prompts will be delivered, and the participant is taken to a new screen with this microintervention prompt on the screen. This second randomization is defined such that each microintervention prompt is equally likely of being delivered. We remark that this second randomization means that a small portion of microinterventions (~10%) may be received more than once. An alternative approach would be to randomize without replacement to ensure a microintervention is delivered only once. We opted for the simpler approach of randomization with replacement, since we do not know whether a microintervention is more effective if delivered more than once, by way of reinforcing a behavior or thought, or less effective because of the redundancy.

### Mobile App

#### Overview

**Figure 2:**
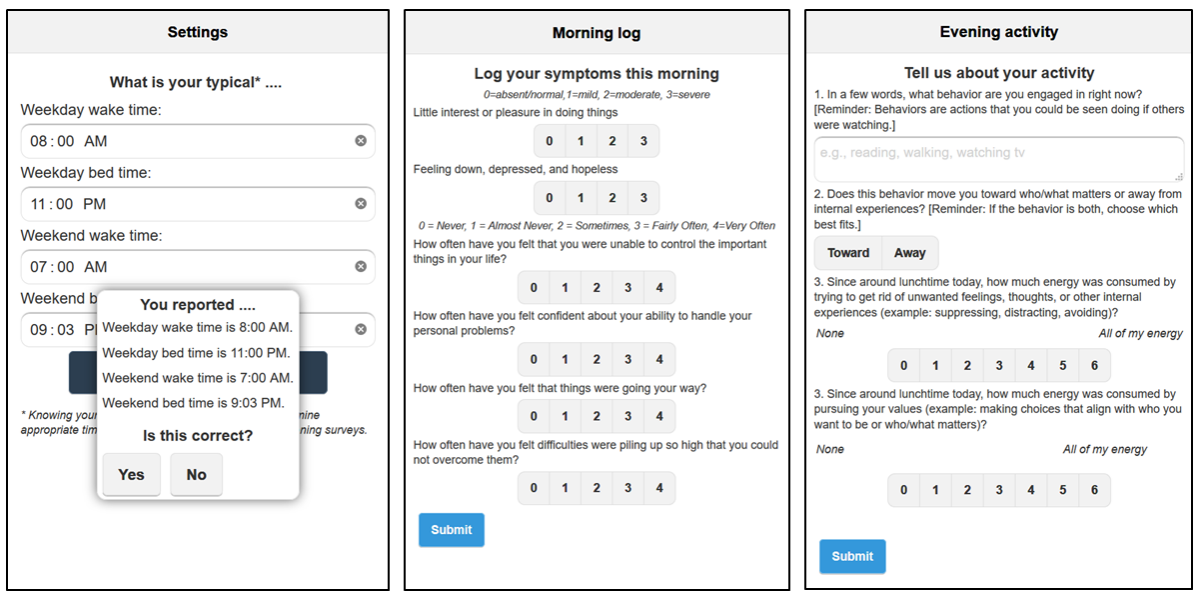
Log functions of the Lorevimo app. The first screen (left) is where participants can set regular weekday and weekend wake times and bedtimes, which determines when they are prompted to log symptoms and activities. The second screen (center) is the mood symptoms log, including depression symptoms and perceived stress. The third screen (right) is the activity questionnaire.

The microintervention is delivered by an app called Lorevimo (see Figure 2), originally developed and tested for assessing engagement strategies for digital self-monitoring of symptoms in bipolar disorder [40,41]. The app was adapted to measure relevant symptoms for each cohort, deliver the microintervention, and integrate responses into the ACT matrix. Lorevimo is restricted to participants through a coded username and password provided to them through the study to protect privacy. The app is available for free in Android through Google Play and in iOS through iTunes. The app was developed using third-party software called Appery.io (Exadel, Inc), which combines drag-and-drop functionality with JavaScript to allow for easy development and advanced control. Appery.io also provides back-end functionality for the app (eg, servers, databases, application programming interface integration, and push notifications) and packages apps for Android and iOS. Lorevimo’s name is derived from its three functions—*log*, *review*, and *visualize your mood*—which we proceed to detail.

#### Log

Once in the morning and evening, participants log symptoms upon clicking *Log* from the home page. After logging, the app randomizes participants to the microintervention. The app presents an ACT-based question to participants randomized to the intervention. Morning and evening are defined based on reported typical wake times and bedtimes on weekdays and weekends, reported at first log-in. Times can be changed under *Settings* in the app. Morning is defined as 2-7 hours after typical wake time. Evening is defined as 3 hours before, to 2 hours after, typical bedtime. Push notifications are sent at 2-hour intervals if symptoms have not been logged within the relevant interval and if the notification can be sent before 30 minutes of the typical bedtime.

#### Review

**Figure 3:**
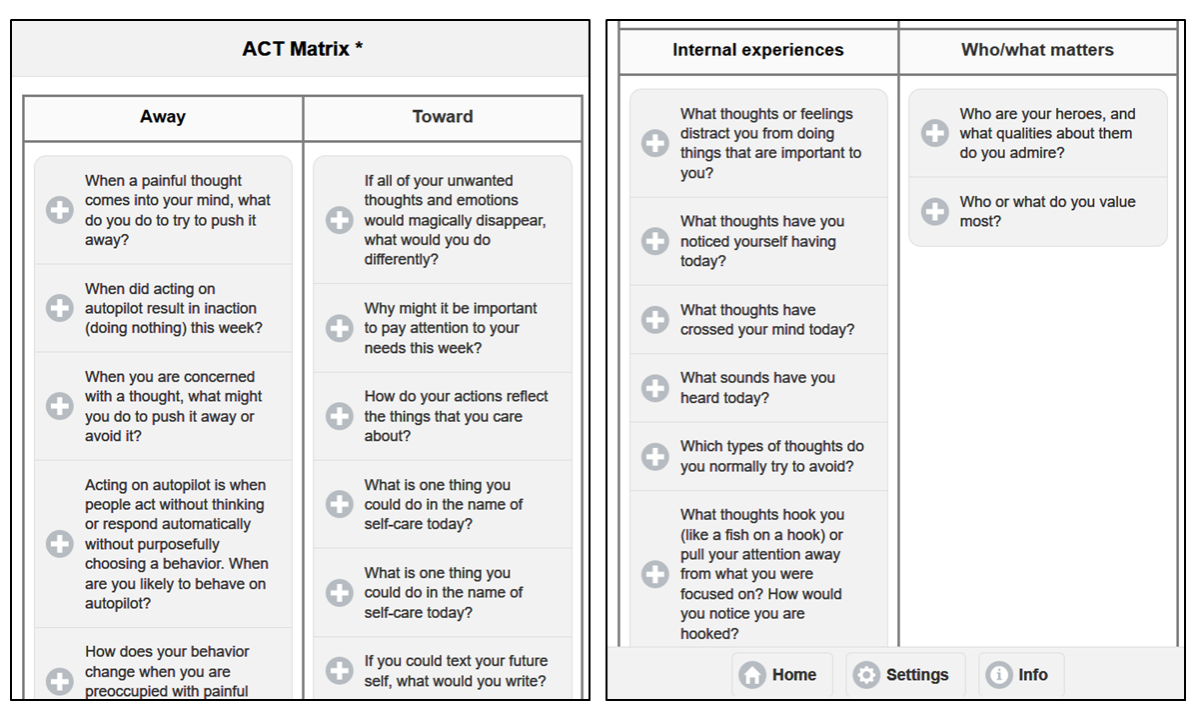
Review function of the Lorevimo app. The first image (left) represents the top half of the acceptance and commitment therapy (ACT) matrix, which sorts the function of behaviors. The second image (right) represents the bottom half of the ACT matrix, which sorts internal experiences and values (ie, who or what matters).

Upon clicking *Review* (see Figure 3) from the home page, the user views an ACT matrix populated with microintervention-completed prompts and responses, sorted based on the targeted concept: avoidance behaviors, values-based behaviors, internal experiences, and values.

#### Visualize

**Figure 4:**
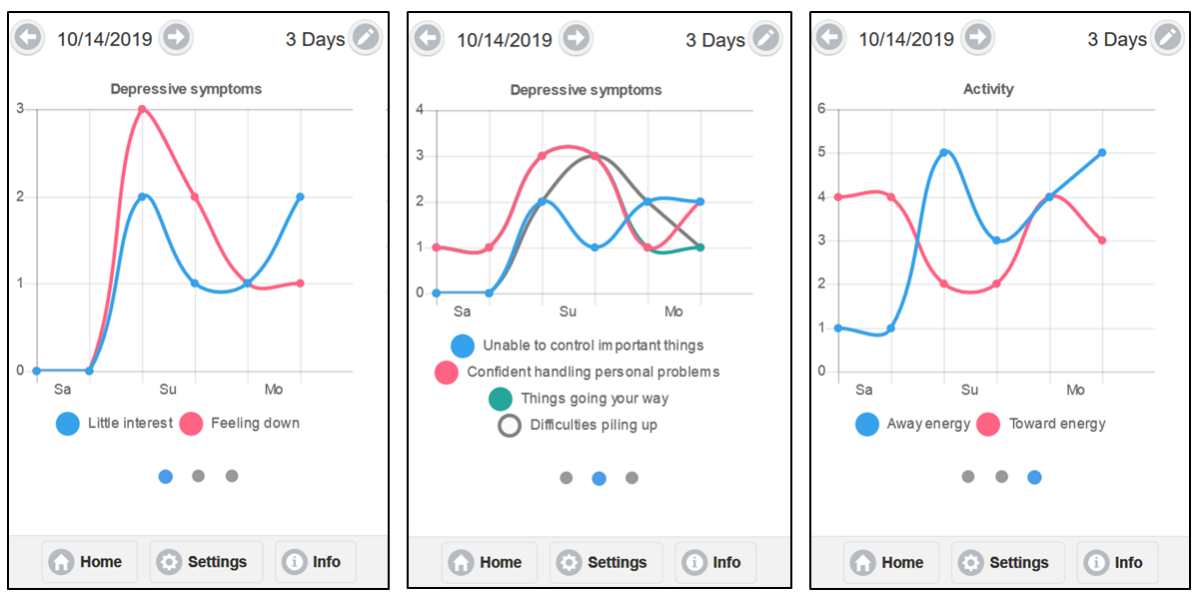
Visualize function of the Lorevimo app. The images represent screenshots of the Lorevimo app’s Visualize function. The first image (left) is a representation of the depressive symptoms in a 3-day (twice daily) interval. The second image (center) conveys the perceived stress symptoms (also a 3-day interval). The final image (right) reflects the responses to the question about energy consumed by avoidance behaviors (ie, away from internal experiences) or values-based behaviors (ie, toward who or what matters).

Upon clicking *Visualize* (see Figure 4) from the app’s home page, the user is shown a graph of symptoms over the past week. The time interval can be changed (ie, 3, 7, or 28 days). The *Visualize* and *Review* functions were designed to help increase awareness, which is a central tenet of ACT, and encourage individuals to continue to log symptoms.

### Microintervention

In order to understand the twice-daily assessment questions regarding behavior and the function of behavior, participants must be familiar with how values, behaviors, and internal experiences fit into the framework of ACT. When participants log in to the app for the first time, they are prompted to watch an introductory video. The same introductory video is used in both studies. In the video, two members of the research team role-play a therapist and client. The purpose of the video is to illustrate the ACT matrix (see Figure 1): identifying and sorting values, internal experiences, avoidance behaviors, and values-based behaviors [23]. The team prompts the viewers to create a matrix reflective of their experiences. The video is intended to promote mindful behavioral awareness and to model the noticing and sorting of one’s experiences, and participants are explicitly told this. The assessment questions are explained in the video as well: form of behavior, function, and the amount of energy expended in avoidance behaviors and values-based behaviors. Answers to microintervention prompts are also used to populate the ACT matrix in the study app. As answers to microintervention prompts accumulate in the matrix, participants can review (see Figure 3) experiences within the theoretical framework.

**Figure 5:**
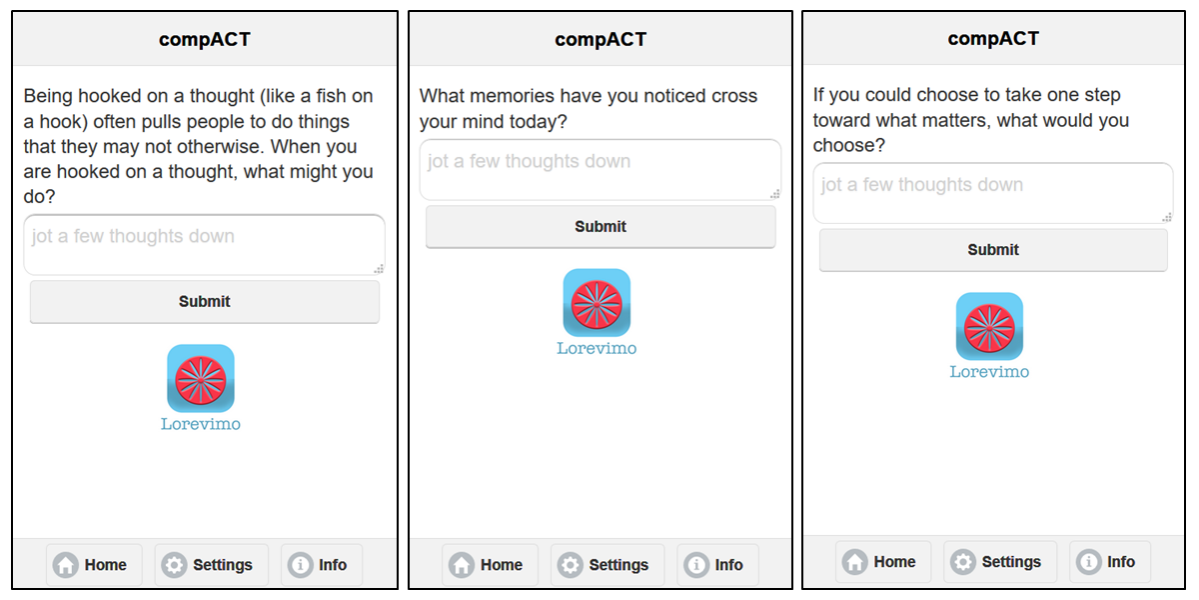
Microintervention examples from the Lorevimo app. These images reflect three of the 84 acceptance and commitment therapy (ACT)-based intervention questions, also allowing space for participants to enter a response. The first (left) is an *openness* question, the second (center) an *awareness* question, and the third (right) an *engagement* question. CompACT: Comprehensive Assessment of Acceptance and Commitment Therapy Processes.

Following completion of the assessment, the participants may be randomized to receive an intervention prompt. The microintervention consists of 84 ACT-based questions developed by the research team. The questions are intended to be small-scale opportunities to build ACT skills, including openness to internal experiences via acceptance and defusion; awareness via mindfulness, self-as-context, and perspective taking; and engagement via values clarification and committed action. These questions can be organized into three subcategories (28 questions per subcategory), each corresponding to a core concept of ACT:

1. Openness to internal experiences and willingness to feel emotions in service of values (see Figure 5, left).
2. Awareness of one's internal experiences (eg, thoughts, emotions, memories, urges, and physical sensations) and external context, as well as awareness of being present and in the moment rather than acting on autopilot (see Figure 5, center).
3. Engagement with values (ie, important people, important areas of life or things, and qualities one wishes to embody) (see Figure 5, right).

The questions are designed to encourage participants to be intentional in action and mindful of thoughts and emotions. The openness subcategory encourages participants to accept internal experiences—positive or negative—rather than engaging in avoidance to suppress or attempt to be rid of such experiences. Awareness questions encourage participants to pay attention on purpose, engaging in mindfulness and intentional presence. Finally, engagement questions prompt participants to consider values: people, things, and qualities of being that are important. In addition, engagement prompts participants to examine the consistency between identified values and current behavior, and to help participants align current behavior with values.

### Study 1: Distressed First-Generation College Students

#### Motivation

The transition to college is associated with changes in health behaviors and mental health functioning, with 50% of college students meeting criteria for a psychiatric disorder [42]. First-generation college students may be at elevated risk for stress and mental health difficulties [43]. The development of adaptive interventions may help provide adequate and accessible care during these dynamic, transitional years. Delivery of these interventions via acceptable and feasible modalities for this population is of utmost importance so that utilization and engagement are prioritized.

This investigation seeks to integrate provision of psychotherapy skills, daily assessment of symptoms and activities, and digital delivery of interventions in a sample of distressed first-generation college students. Given that college students are often required to utilize technology in the classroom, as well as potentially in other settings (eg, using public transportation on campus), the development of an app aimed to reduce distress and increase values-based behaviors may have wide-ranging impact on the well-being of college students, while also integrating into a familiar digital context.

#### Participants

A total of 50 students will be recruited from the University of Wisconsin–Madison to participant in a 6-week study focusing on effectiveness, safety, and feasibility of the microintervention. This study has been approved by the Health Sciences Institutional Review Board at the University of Wisconsin–Madison (2019-0819). Inclusion criteria, which will be determined from a screening instrument, are that participants must (1) be currently enrolled freshman or sophomore students at the University of Wisconsin–Madison, (2) be first-generation college students (ie, neither parent or legal guardian of the student holds a bachelor’s degree or above), (3) endorse functional impairment by distress at the time of screening, defined as experiencing impairment in one or more domains on at least 4 out of the past 7 days, and (4) have a smartphone. There were no exclusion criteria. Participation is open to all gender identities, adults aged 18 and 19 years, and all ethnic and racial groups. A consent discussion will occur by phone, followed by electronic submission of signed consent through Research Electronic Data Capture (REDCap) (Vanderbilt University). A PDF of the informed consent document will be provided for participants’ records via email. Recruitment began in fall 2019. Recruitment methods will include mass email, posting flyers on the University of Wisconsin–Madison campus, and brief presentations during University of Wisconsin–Madison classes. Subject recruitment and enrollment will occur over 18 months with the total duration of the trial expected to be 30 months, including the microintervention and follow-up data collection.

#### Remuneration

Participants are remunerated for both research and intervention activities. Participants are remunerated for each week in which they complete at least 50% of daily in-app assessments throughout the 6-week intervention period, as well as for completion of baseline and follow-up assessments. A bonus will be provided to participants who complete all 6 weeks of the intervention period.

#### Assessments

Assessments measure stress and depression. Participants are assessed in REDCap at baseline, exit, 3-month follow-up, and 6-month follow-up with the Perceived Stress Scale 10 (PSS-10) [32] and the Patient Health Questionnaire 9 (PHQ-9) [33]. The Patient Health Questionnaire 2 (PHQ-2) consists of the first two items from the PHQ-9, measuring the two gatekeeper symptoms of depression: dysphoria and anhedonia. Twice-daily in-app assessments include the Perceived Stress Scale 4 (PSS-4) [44], the PHQ-2, and the ACT Activity Survey.

#### Outcomes

Primary outcomes for the college student cohort measure effectiveness, feasibility, and safety of the microintervention (see Table 1):

1. Effectiveness. Because the microintervention is delivered immediately after an assessment, outcomes to assess effectiveness are based on the very next assessment. That is, if the intervention is provided in the morning, then its effect is measured on assessments in the evening. Responses are called *proximal outcomes* for their proximity to the intervention. Proximal outcomes of interest are primarily responses to the last two questions on an ACT Activity Survey, but we will also consider scores on symptom scales (ie, PHQ-2 and PSS-4).
2. Feasibility. One potential barrier for a micro-randomized trial is insufficient proximal outcome data for measuring effectiveness. Given proximal outcomes are recovered from in-app assessments, then feasibility outcomes include adherence to in-app assessments (ie, completion of at least 50% of daily items). A similar 50% benchmark was used in our preceding study [40,41]. Participants can respond to 20 items each day ([6 symptom items + 4 activity items] × 2 per day). Additional outcomes include quality of engagement with the ACT microintervention (ie, number of words in responses and relevance of content).
3. Safety. While safety outcomes are often related to adverse events, given an expected minimal risk of this study, we focused on safety outcomes that indicate a worsening of symptoms. Specifically, the proportion of individuals with PHQ-9 scores above 10 between baseline, exit, and follow-up will be examined. These outcomes provide low-level evidence (ie, not causal evidence) that participating in the study worsens mood symptoms.

#### Power Analyses

For the college student cohort, power was calculated using the supported calculator for adaptive interventions [26], available online [45]. In this case, sample size was calculated to test for a linear effect of time of intervention on proximal outcome measures (ie, depression, stress, and activity). We expect a smaller effect size, on average, than an effect size for an in-person psychotherapy intervention [26]. We expect a small-to-medium effect size (~0.1-0.2) on average. In addition, we hypothesize that participants will respond to 80% of prompts based on a prior study of user engagement that used Lorevimo [41]. Conservatively assuming a linear effect, which on average produces a small or medium effect size of 0.1, and assuming subjects respond 80% of the time, 50 subjects will yield 83.8% power to detect a linear effect with a significance level of .05.

#### Statistical Analyses

For feasibility, our hypothesis is that participants will adhere to in-app assessments (ie, respond to over half of the assessments per day, for over 60% of the days of the intervention period on average). To test this hypothesis, a one-sample z test of proportions will be performed to determine whether average percent of days to which a participant adheres to in-app assessments differs significantly from 60%. Given our overarching goal of a micro-randomized trial in a transdiagnostic population, a cutoff of 60% is considered a lower bound for adherence needed to power such a larger study (ie, go or no-go criteria) and is thought to be a meaningful deviation from the 80% adherence from our preceding study. For reference, 117 individuals would be needed if individuals only responded to 30% of prompts (60% of days × half the completion) to achieve the same power as in this study under the same assumptions as above. Our hypothesis related to safety is that the proportion of individuals with a PHQ-9 score of 10 or above between baseline to exit or between baseline to each follow-up will not increase, and a one-sample z test of proportions will be performed to determine whether these proportions are significantly different from zero.

To test effectiveness, we will use a weighted and centered least squares method [29,46] to estimate and test the average effect of delivering a microintervention on each proximal outcome as a function of time in the study conditional on the participant being available for randomization (ie, the participant completed the in-app assessment at the prior time point). Two proximal outcomes will be evaluated: energy devoted to values-based or avoidance behaviors, as measured on our ACT Activity Survey. The treatment effect model will control for an intercept and time and will be linear in the binary intervention variable, centered at the probability of receiving an intervention, and the interaction (ie, time × centered intervention variable). To account for repeated samples and nonindependence, robust standard errors will be calculated using a sandwich estimator [46]. The interaction term will be tested for statistical significance (*P*<.05).

Data may be missing if a participant does not complete an in-app assessment, resulting in missing proximal outcomes. This missingness may introduce estimation bias for average microintervention effects if the microintervention delivery were to influence whether or not the next proximal outcome is missing. If no more than 10% of the data are missing, then our primary analysis would be a complete-case analysis, which assumes that whether or not the proximal outcome is missing is independent from microintervention delivery and includes only data points without a missing proximal outcome in our analysis. Follow-up analysis would then examine the sensitivity of our estimates to this independence assumption in two steps. First, we would identify variables available prior to each randomization that predict whether or not the proximal outcome is missing. Candidate variables include the outcome of interest measured at prior time points, prior number of interventions and assessments, and prior number of missing proximal outcomes and assessments. Second, we would then repeat analyses controlling for these variables in the weighted and centered least squares method. If more than 10% of the data are missing, then the above analysis, which controls for the variables’ associated missingness, will be used as our primary analysis, and a complete-case analysis would be used as a follow-up analysis.

#### Exploratory Analyses

To better optimize the intervention, further exploratory analyses will be performed to determine if the effect of the intervention on proximal outcomes is moderated by momentary information, such as the microintervention subcategory (ie, engagement, openness, or activity) and current symptoms and behavior. Additional exploratory variables of interest include childhood trauma and resilience, measured via self-report.

We will perform a qualitative analysis of microintervention responses to examine comprehension of ACT processes. Two members of the research team (SH and AV), who were trained in identifying and applying the theoretical components of ACT, will code responses for process alignment. Each microintervention prompt targets one of the three core ACT processes described above: openness, awareness, and engagement. The methods for developing codes and results of this analysis will be published at a later date. Coders will also code behavioral responses and the function of behavior. In particular, we are interested in whether behaviors of the same form (eg, exercise) serve different functions throughout the study (eg, avoidance of stress vs pursuit of health). This diversity of function would be indicative of behavioral awareness.

### Study 2: Individuals With Bipolar Disorder

#### Motivation

Bipolar disorder is a chronic mood disorder that affects 2.4% of individuals worldwide [47] and ranks seventh among disability-causing diseases among men and eighth among women [11]. Individuals with bipolar disorder experience profound shifts in mood ranging from depression to mania. Treatment includes medication and/or psychotherapy. However, relapse and nonadherence with medication, along with access to care, remain common barriers to maintaining stability in mood. Consequently, mood may shift dramatically within days, with little advanced warning, and due to unpredictable events [48]. Treatment guidelines are often insufficiently nuanced to predict when, where, and how to intervene. New adaptive strategies are necessary to optimize promising psychotherapies in an effort to make them more accessible and efficient at interpreting individual needs. The current micro-randomized trial based on ACT takes a first step toward investigating effective mobile adaptive interventions for bipolar disorder.

#### Participants

A total of 30 participants will be recruited from the Prechter Longitudinal Study of Bipolar Disorder [49] to participate in a 6-week study examining the safety and feasibility of the microintervention. The institutional review boards at the University of Michigan (HUM126732) and University of Wisconsin (2017-1322) have approved the study. The study did not include a data safety monitoring board. Inclusion criteria include a diagnosis of bipolar disorder (ie, type I, type II, or not otherwise specified), agreement to be contacted for future research, and access to a smartphone. Participants in the Prechter Longitudinal Study of Bipolar Disorder have completed a Diagnostic Interview for Genetic Studies (DIGS) to collect mental and physical health history, including bipolar disorder diagnosis. Potential participants will be contacted via recruitment email or phone call. If interested and eligible, participants will consent by phone, and the consent form will be electronically signed through the Health Insurance Portability and Accountability Act (HIPAA)-compliant data capture software REDCap. Adults of all genders and ethnic and racial backgrounds are eligible. Following a preceding study of digital self-monitoring in bipolar disorder [40,41], participants will be mailed an activity tracker: Fitbit Alta HR. The inclusion of a Fitbit would allow us to explore a possible relationship between mobile ACT effectiveness and sleep, activity, and heart rate, which are considered to both indicate and moderate symptoms of mania and depression. Participants will contact the study team upon receipt, and an entrance interview will be completed.

#### Remuneration

Participants are remunerated based on research activities, defined as the completion of exit and entrance interviews and completion of participation in study weeks 1 through 5. Remuneration is submitted once participants complete the exit interview at the end of the 6 weeks. If a participant ends their participation in the study early, they are to be remunerated based on how many weeks they have completed, and whether or not they completed the exit interview.

#### Assessments

Assessments for the bipolar cohort focus on manic and depressive symptoms (see Table 1). Participants are assessed over the phone at baseline and exit with the Young Mania Rating Scale (YMRS) [34] and the Structured Interview Guide for the Hamilton Depression Rating Scale (SIGH-D) [35]. The 36-Item Short Form Survey (SF-36) [36] is administered at these time points to assess general health and well-being. Shortened versions of the YMRS and the SIGH-D are completed via twice-daily in-app assessments. These shortened versions were first introduced in Cochran et al [49] in a study of engagement in digital self-monitoring among individuals with bipolar disorder. While the validation of psychometric properties of these shortened versions is ongoing, they were introduced in an effort to address a need for a digital instrument that is brief but can separately measure severity of manic symptoms and severity of depressive symptoms. The same ACT Activity Survey used in the college sample will be assessed in-app. The ACT Activity Survey consists of four questions that target ACT concepts: (1) What behavior are you engaging in right now? (2) Is this behavior moving you toward who/what matters or away from internal experiences? (3) Since this [morning or lunch time], how much energy was consumed by avoidance? (4) Since this [morning or lunch time], how much energy was consumed by pursuing values? See the assessment in Multimedia Appendix 1.

#### Outcomes

Primary outcomes for the bipolar cohort measure feasibility and safety of the microintervention (see Table 1). Effectiveness is left as a secondary outcome due to limited power and will be used to determine power for a future study.

1. Feasibility. Following the other sample, outcomes to assess feasibility are adherence to in-app assessments (ie, completion of at least 50% of daily items).
2. Safety. Safety outcomes are average changes in YMRS and SIGH-D scores from baseline to exit, proportion of individuals with increased YMRS scores from baseline to exit, and proportion of individuals with increased SIGH-D scores from baseline to exit. These outcomes provide low-level evidence (ie, not causal evidence) that participating in the study is worsening mood symptoms.
3. Effectiveness (secondary outcome). Proximal outcomes of interest are primarily responses to the last two questions on an ACT Activity Survey, but we will also consider scores on symptom scales (ie, in-app manic and depressive symptom assessments).

#### Power Analyses

A sample size of 30 subjects would yield 58.6% power to detect a linear effect of microinterventions that is, on average, 0.1 over the study, assuming subjects respond 80% of the time, with a significance level of .05. However, the sample size for the bipolar cohort was not specified to have sufficient power to evaluate effectiveness, since effectiveness of the microintervention is not a primary outcome. The sample size for the bipolar cohort was specified to estimate the intervention effect and adherence to in-app assessments to use as input for powering a larger study.

#### Statistical Analyses

Our feasibility hypothesis, which is identical to the feasibility hypothesis for the college sample, is that participants will adhere to in-app assessments (ie, respond to over half of the assessments per day, for over 60% of the days of the intervention period on average). To test this hypothesis, a one-sample z test of proportions will be performed to determine whether average percent of days to which a participant adheres to in-app assessments differs significantly from 60%. Our safety hypotheses are that mean YMRS or SIGH-D scores will not decrease from baseline to study exit, an equal proportion of individuals will see an increase in YMRS scores as a decrease from baseline to study exit, and an equal proportion of individuals will see an increase in SIGH-D scores as a decrease from baseline to study exit. A one-sample *t* test will be performed to determine whether changes in mean scores are significantly different from zero, and a rank test will be performed to test for equal proportions. Our effectiveness hypothesis is that the microintervention has an approximate linear effect in time on proximal outcomes of energy devoted to values-based or avoidance behaviors. As for the college sample, the weighted and centered least squares method [29,46] will be used to estimate and test the average effect of delivering a microintervention on each proximal outcome as a function of time in the study, conditional on the participant being available for randomization (ie, the participant completed the in-app assessment at the prior time point). The weighted and centered least squares method will control for the intercept and time and will use a linear treatment effect model with a binary intervention variable, centered at the probability of receiving an intervention, and interaction (ie, time × centered intervention). Robust standard errors will be calculated [46]. The interaction term will be tested for statistical significance (*P*<.05) to determine if the invention has a linear effect on proximal outcomes. Missing data for the bipolar sample would be handled in the same way they are handled for the college sample.

#### Exploratory Analyses

For the bipolar sample, we will also explore whether the effect of the microintervention on proximal outcomes is moderated by momentary information, such as sleep duration, heart rate variability, and current symptoms of depression and mania. Additionally, we will perform a qualitative analysis of responses to examine comprehension of ACT processes, using an identical coding process described in the Exploratory Analyses section for Study 1. By qualitatively coding responses from both samples, we would also assess how each sample may differ in how participants comprehend and engage in ACT processes.

## Results

The study app was released to Google Play and iTunes in fall 2019 and is password protected to restrict use to study participants. Recruitment for the bipolar sample began on September 10, 2019. As of November 16, 2019, we had 10 people enrolled and consented, and participation in the study began on September 13, 2019. Recruitment for the college sample began on October 5, 2019. As of November 16, 2019, 223 participants have completed the screening survey, with 39 being eligible. As of November 16, 2019, we had 14 people enrolled and consented in the study, and participation in the study began on October 18, 2019.

## Discussion

### Overview

These investigations seek to evaluate the feasibility, safety, and effectiveness of optimizing mobile-based microinterventions among two cohorts: a sample of individuals with bipolar disorder and a sample of distressed first-generation college students. The studies address important public health concerns, including large treatment gaps that leave many suffering from psychiatric disease untreated [11], treatments that may be perceived as inaccessible or incompatible with other life demands, and inadequate proximal assessment of symptom changes directly after intervention. The microintervention design allows for the examination of proximal change in symptoms in the assessment just hours after the intervention was delivered, which presents a distinct advantage when compared to traditional RCTs. Further, the delivery of a microintervention via smartphone meets participants in a familiar environment optimized for on-the-go use. Participants can self-tailor usage to seek additional support when needed, reviewing symptoms or visualizing previously input content. The examinations in this study offer a potentially accessible tool that could reasonably be implemented in future studies among rural samples lacking access to care, among samples with chronic disease or other barriers preventing attendance at weekly psychotherapy, or as an adjunct to brief in-person interventions. As such, the studies will fill critical gaps in the current literature and provide information to be utilized in future studies.

These studies seek to examine a transdiagnostic approach in two different samples in order to determine whether such an intervention is applicable and feasible among individuals of different demographic characteristics and psychiatric symptom profiles. Such an approach is important to consider, given that widely differing psychotherapy approaches for specific psychiatric disorders create further barriers to psychiatric care, given inadequate training, dissemination, and implementation, so that patients can access treatment. Should ACT demonstrate feasibility and preliminary effectiveness, a large RCT applying ACT across diagnoses and demographics would be indicated.

### Limitations

Any findings from these studies should be considered in light of several limitations. First, though we designed mobile ACT to support psychological or pharmacological treatment, we will not collect data regarding psychological or pharmacological treatment. Thus, we will not be able to determine if current and prior treatment modifies the effect of mobile ACT. Second, usage data are not being collected beyond logging of symptoms, so time devoted to reviewing or visualizing symptoms will not be examined. The content and word count of responses to the microintervention questions will be tracked, however. Word count alone may not be indicative of engagement quality, which we will explore further with a qualitative analysis. Data collected regarding completion of the introductory ACT video are limited to the average time all participants spent watching the video, the percentage of viewers who watched until the end, and the total number of views. Individual-level data will not be collected, and we will be unable to verify whether participants watched the video and/or completed it in its entirety before using the study app. Furthermore, some of the content may be most effective when viewed in combination with, or subsequent to, other content, and given the randomized nature of the interventions, sequenced interventions are not offered. Nevertheless, we will evaluate the proximal impact of the microinterventions individually, bearing on the question as to whether small-scale interventions are impactful in isolation, both in terms of mood and activity.

Among the bipolar cohort alone, we will have insufficient data to draw conclusions about effectiveness and, as such, the only conclusions drawn will be regarding safety and feasibility. The results will be considered as future studies are designed. In addition, the bipolar cohort received Fitbit activity trackers as part of the study, and the act of wearing a Fitbit may prompt behavioral change in physical activity or sleep. Among the college student cohort, first-generation college student status is self-reported and not verified in order to protect participant privacy. Furthermore, depressive symptoms are measured with a self-report scale and, as such, no diagnostic conclusions can be drawn. The activity measure was developed for this study specifically and is not yet validated, though authors intend to examine the psychometric properties of the scale. Additionally, awareness of avoidance and values-based behaviors may change throughout the intervention, and one of the goals of the interventions is to increase mindful awareness. Similarly, the in-app assessments of manic and depressive symptoms via shortened versions of the YMRS and the SIGH-D have yet to be validated. Additionally, participants in the college student cohort receive remuneration on a weekly basis, granted they complete at least 50% of study activities (ie, respond to at least seven of the 14 daily prompts from the study app). This incentive to participate may result in increased adherence rates, limiting us from generalizing our findings regarding the feasibility of the intervention to a real-world setting.

## Appendix

Multimedia Appendix 1 Summary statement from the National Institutes of Health (NIH) K01 grant (principal investigator: AC). The presented protocol for the bipolar sample is based on Aim 3 of this NIH K01 grant.

## Abbreviations

ACT: acceptance and commitment therapy
DIGS: Diagnostic Interview for Genetic Studies
GSK: GlaxoSmithKline
HIPAA: Health Insurance Portability and Accountability Act
NIH: National Institutes of Health
PHQ-2: Patient Health Questionnaire 2
PHQ-9: Patient Health Questionnaire 9
PSS-4: Perceived Stress Scale 4
PSS-10: Perceived Stress Scale 10
RCT: randomized controlled trial
REDCap: Research Electronic Data Capture
SF-36: 36-Item Short Form Survey
SIGH-D: Structured Interview Guide for the Hamilton Depression Rating Scale
YMRS: Young Mania Rating Scale
