# Supplementary material for: Optimizing an Acceptance and Commitment Therapy Microintervention Via a Mobile App With Two Cohorts: Protocol for Micro-Randomized Trials
Source: JMIR Res Protoc. 2020 Sep 23;9(9):e17086. doi: 10.2196/17086 (PMC7542401; doi:10.2196/17086)
Supplement: Multimedia Appendix 1 [file resprot_v9i9e17086_app1.pdf]

SUMMARY STATEMENT

PROGRAM CONTACT:
Mark Chavez
301-443-8942
mchavez1@mail.nih.gov

(Privileged Communication)

Release Date: **09/13/2016**
Revised Date:

Application Number: **1 K01 MH112876-01**

Principal Investigator

COCHRAN, AMY LOUISE

Applicant Organization: UNIVERSITY OF MICHIGAN

Review Group: **ZMH1 ERB-D (02)**
National Institute of Mental Health Special Emphasis Panel
Interventions/Biomarkers

Meeting Date: **09/09/2016**
Council: **JAN 2017**
Requested Start: **04/01/2017**

RFA/PA: **PA16-190**
PCC: **AK-TN**

Project Title: **Modeling mood course to detect markers for effective adaptive interventions**

SRG Action: **Impact Score:20**
Next Steps: **Visit http://grants.nih.gov/grants/next_steps.htm**
Human Subjects: 30-Human subjects involved - Certified, no SRG concerns
Animal Subjects: 10-No live vertebrate animals involved for competing appl.
Gender: 1A-Both genders, scientifically acceptable
Minority: 1A-Minorities and non-minorities, scientifically acceptable
Children: 3A-No children included, scientifically acceptable
Clinical Research - not NIH-defined Phase III Trial

Project Year	Direct Costs Requested	Estimated Total Cost
1	124,738	134,717
2	126,982	137,140
3	129,290	139,633
4	131,681	142,215
TOTAL	512,691	553,706

ADMINISTRATIVE BUDGET NOTE: The budget shown is the requested budget and has not been adjusted to reflect any recommendations made by reviewers. If an award is planned, the costs will be calculated by Institute grants management staff based on the recommendations outlined below in the COMMITTEE BUDGET RECOMMENDATIONS section.

1K01MH112876-01 Cochran, Amy

RESUME AND SUMMARY OF DISCUSSION: This application for a Research Scientist Development Award is from a candidate interested in utilizing computational methods on highly dimensional data to improve the long term monitoring and ultimately the treatment of patients with bipolar disorder. The candidate has assembled an exceptional group of mentors that can add to her already exceptionally strong background in mathematics with training is biostatistics and mental disorders. The additional training described is designed to allow her to develop modeling approaches that can accurately characterize and test markers of mood course and sleep over time in bipolar illness. The model's predictive strength and feasibility of using it to fit a psychosocial intervention, a clinical phone call, into an adaptive intervention would be tested in a series of micro-randomized clinical trials. The over-arching goal is to be able to provide the right intervention to the right person at the right time in order to optimize wellness. Overall, reviewers felt that this application is outstanding with notable strengths in the candidate herself, the mentoring, the career development plan and institutional support. The candidate's potential to be a significant contributor to the field was noted. A few areas were noted that might be improved upon. These included additional attention to the technical and feasibility aspects of using actigraphy, a more thorough review of the literature that has used similar approaches within biostatistics to select and time best treatments, and additional attention to the types of data analysis issues that arise in clinical trials such as non-adherence and missing data.

DESCRIPTION (provided by applicant): Bipolar (BP) disorder is a chronic illness of profound shifts in mood ranging from mania to depression. BP is successfully treated by combining medication with psychosocial therapy, but care can prove inadequate in practice. With gaps in coverage and medication, along with imprecise guidelines on when, where, and how to intervene, promising psychosocial therapies require adaptive strategies to better address the specific needs of individuals in a timely manner (NIMH Strategy 2). To accomplish this, however, requires evidence-based practices for adapting a psychosocial therapy. This Mentored Research Scientist Development Award aims to address this knowledge gap, by (1) establishing a mobile health platform for translating a psychosocial therapy in BP into an effective adaptive intervention and (2) facilitating the transition of a junior researcher, at the interface of mathematics and psychiatry, into an independent researcher of effective adaptive interventions. The research effort is founded on a mobile health platform that combines evidence-based markers of mood for long-term monitoring with a micro-randomized trial, designed for optimizing mobile health adaptive interventions. In Aim 1, we use modeling to characterize and test new markers of mood course that account for volatility, a feature that masks effects of a therapy on mood. In Aim 2, we explore the potential for long-term monitoring of BP with interpretable markers from actigraphy. In Aim 3, best practices from Aim 1 and 2 are integrated with a micro-randomized trial into a mobile health platform. We then test the feasibility of using the platform to translate a psychosocial therapy, clinical phone call, into an adaptive intervention. If successful, this work will advance the Candidate's independent goal of adaptive scheduling of phone-calls with BP individuals. To complement the research agenda, the award will expand the Candidate's background in Computational Psychiatry into the area of Translational Psychiatry by providing training in five strategic areas: (1) clinical assessments, (2) psychosocial therapy, (3) mobile health interventions, (4) adaptive clinical trials, and (5) open-access scheduling. Dr. Melvin McInnis, Thomas B and Nancy Upjohn Woodworth Professor of Bipolar Disorder and Depression and Professor of Psychiatry at the University of Michigan, will be the primary mentor and will guide clinical aspects of the training (Training Objectives 1–3); Dr. Amy Kilbourne, Professor of Psychiatry and Acting Director of VA/HSR&D's Quality Enhancement Research Initiative, will guide training in the application of adaptive trial designs that involve psychosocial therapy (Training Objectives 2,4–5); and Dr. Susan Murphy, Herbert E. Robbins Distinguished Professor of Statistics, will guide training into methodology for adaptive trial design and mobile health interventions (Training Objectives 3–4). The proposed K01 award promises to train a junior scholar to address technically-challenging problems in mental health. This work is aligned with the NIH and NIMH missions of providing precise clinical care.

PUBLIC HEALTH RELEVANCE: Bipolar disorder, a chronic disease of profound shifts in mood, is ranked as the seventh and eighth highest cause of disability among male and female adults by the World Health Organization. The aim of this project is to measure mood course in the moment, over time, in individuals, thus directing therapy to individuals when it is most needed. If successful, this project will (1) establish a mobile health platform for general use in developing effective adaptive interventions in bipolar disorder and (2) facilitate the transition of a junior researcher, at the interface of mathematics and psychiatry, into an independent researcher of effective adaptive interventions.

CRITIQUE 1:

Candidate: 1
Career Development Plan/Career Goals & Objectives: 1
Research Plan: 2
Mentor/Co-Mentor(s), Consultant(s), and Collaborator(s): 1
Environment and Institutional Commitment to the Candidate: 1

Overall Impact:
This is an excellent, well-written and innovative application from a talented applicant with technical and mathematical skills who will enhance her training in applied clinical issues. The project promises to improve quantification and prediction of mood states relevant for bipolar disorder on a dimensional scale. Actigraphy will be used to enhance predictive accuracy. It has potential to guide clinical intervention for the right person at the right time, and micro-randomized trials will provide evidence-based data in this regard.

1. Candidate:
Strengths
- Excellent background in both mathematics and medical applications which will be of great utility for making progress in computational psychiatry.
- Introduced novel, principled and sophisticated methodology for classifying bipolar disorder using Bayesian non-parametric hierarchical models based on mood data.
- Successfully used modeling and statistics for other related projects in the domains of medicine and health, including large scale mobile data to quantify and predict sleep habits around the world, and modeling uterine contractions.

Weaknesses
- [None noted]

2. Career Development Plan/Career Goals & Objectives/Plan to Provide Mentoring:
Strengths
- Training will enhance the candidate's clinical expertise and skills, including assessments, therapy, and adaptive clinical trials. This will complement the candidate's background in mathematics, statistics and models of mood dynamics so that her work is grounded and informed by clinical considerations.
- Training will also increase knowledge of statistical methods including reinforcement learning applied to adaptive clinical trials

Weaknesses
- [None noted]

3. Research Plan:
Strengths

- Approach has potential to explain and predict whether and when a particular patient will benefit from a particular psychosocial therapy.
- Quantitative classification of BP patients from mood data in way that can handle random changes in mood separately from meaningful changes, is dimensional, and yields clinically relevant sub-samples.
- Innovative approach using surrogate biomarkers which ultimately could improve quantification of mood in bipolar disorder, and potentially be translated for other conditions.
- “Micro-randomized trial” approach, designed by one of the mentors, is promising here and will produce evidence-based knowledge.
- Preliminary data shows feasibility of use of actigraphy.

Weaknesses

- Unclear whether time scale of observations is sufficient to predict long-term outcomes or estimate volatility at longer periods.

4. Mentor(s), Co-Mentor(s), Consultant(s), Collaborator(s):

Strengths

- The mentorship team is excellent and provides appropriate expertise in bipolar disorder, clinical assessments, psychosocial therapy, and statistics with a focus on adaptive clinical trials based on reinforcement learning.

Weaknesses

- [None noted]

5. Environment and Institutional Commitment to the Candidate:

Strengths

- The environment and institutional commitment is excellent.

Weaknesses

- [None noted]

Protections for Human Subjects:

Acceptable Risks and Adequate Protections

- The plan is adequate and thorough and benefits outweigh any risks

Data and Safety Monitoring Plan (Applicable for Clinical Trials Only):

- Acceptable
- The plan is adequate and safety / monitoring will be ensured by the DMSB (board)

Inclusion of Women, Minorities and Children:

- Sex/Gender: Distribution justified scientifically
- Race/Ethnicity: Distribution justified scientifically
- For NIH-Defined Phase III trials, Plans for valid design and analysis: N/A
- Inclusion/Exclusion of Children under 18: Excluding ages <18; justified scientifically

Training in the Responsible Conduct of Research:

- Acceptable

Comments on Format (Required):

- The plan is appropriate, with workshop training and individual mentorship with face-to-face discussion.

Comments on Subject Matter (Required):

- The plan is appropriate and thorough.

Comments on Faculty Participation (Required; not applicable for mid- and senior-career awards):

- The plan is appropriate, as multiple faculty with relevant expertise will be engaged and are enthusiastic supporters of the work.

Comments on Duration (Required):

- The plan is appropriate.

Comments on Frequency (Required):

- The plan is appropriate.

CRITIQUE 2:

Candidate: 1
Career Development Plan/Career Goals & Objectives: 2
Research Plan: 2
Mentor/Co-Mentor(s), Consultant(s), and Collaborator(s): 1
Environment and Institutional Commitment to the Candidate: 1

Overall Impact:
This application is submitted by a highly productive, forward-thinking junior investigator who is very well positioned to be one of the leaders of an emerging field. The candidate has a demonstrated record of focused relevant work and a very strong group of mentors who are highly committed to her success at the University of Michigan. The study plan builds on very recent findings in the area of mHealth in the study of severe psychopathology and is consistent with NIMH strategic objectives and the evolving RDoC framework for conceptualization of dimensions of illness. The staged scientific approach is structured, but flexible enough to accommodate a range of outcomes in each phase. The resources and infrastructure available to the candidate are exceptional and very well suited to support her study plan and long-term objectives. Beyond the individual candidate's growth and development as a researcher, the study findings stand to make an important contribution to the field.

1. Candidate:
Strengths

- Highly accomplished for her professional stage.
- Experienced in multidisciplinary collaboration.
- Intellectually curious and intrinsically driven.
- Clearly understands the need for entrepreneurial activities in science and is active in creating her own solutions rather than wait for them to be made available.
- Demonstrated capacity for self-initiated and self-directed scientific work.
- Exceptionally strong background in quantitative methods and accumulating exposure to clinical research.
- Has already been involved in research directly related to the topics of this application and led manuscript preparation scientific assembly/workgroup/ symposia discussions.

Weaknesses

- N/A

2. Career Development Plan/Career Goals & Objectives/Plan to Provide Mentoring:
Strengths

- Well designed, multidisciplinary mentoring plan.

- Clear and well-articulated career goals that are consistent with an emerging area—the application of mathematical modeling to psychiatric research. The candidate can play an important role in helping shape this evolving field.
- Multi-pronged training model with emphasis on topics that are directly pertinent to the candidate's objectives and to the state of the emerging field—clinical assessment, therapy, mHealth, adaptive clinical trials, and time-sensitive scheduling.

Weaknesses

- The mentoring experience with Dr. Murphy seems to be largely limited to group-based activities. The candidate would benefit from more 1:1 interaction and scheduled check-in and support with Dr. Murphy or less senior members at the statistical reinforcement learning lab.
- University of Michigan offers outstanding mentorship opportunities. Given the candidate's ambition and long-term objectives she could also benefit from short-term on-site and/or distal mentorship experiences with secondary advisors at other institutions. This would help round out her experience and provide even broader context for her work.

3. Research Plan:

Strengths

- Thoughtful application of dimensional metrics derived from a theoretical model of mood in Bipolar disorder.
- Fast-paced but realistic and focused staged approach. Outcomes of each stage inform the following one, but specific directional findings are not essential to proceed.
- Good consideration of potential barriers in modeling in stage 1 and pitfalls in the form of unexpected outcomes in stage 2. This helps prevent major barriers to accomplishing all study aims.
- Pilot work produced preliminary findings that are a solid launching pad for the proposed effort.
- Innovative use of novel trial design.
- Project ends with a feasibility run that paves the way for the next application, led or co-led by the candidate.

Weaknesses

- There is good consideration of adherence to the study protocol, but little consideration of the limitations of actigraphy, and passive sensing more generally, in capturing the constructs of interest in a valid reliable manner.
- Are there additional measures of adherence to actigraphy that can help inform how data are interpreted, in terms of long term viability of the "wearable" approach (e.g., subjective discomfort, % of people wearing it loosely, skin reaction, charging neglect, malfunction, etc.)?
- The feasibility measures are not fully articulated; the primary measures described seem more clinical/ health related than feasibility-focused. How specifically will patients evaluate their experience? Are there go/no-go feasibility outcomes that will be applied to determine the next step in the research?

4. Mentor(s), Co-Mentor(s), Consultant(s), Collaborator(s):

Strengths

- Three outstanding mentors, each with expertise in subareas that are directly relevant to the candidate's professional and sciatic objectives.
- Dr. McInnis is very well situated to serve as primary mentor and supervisor of the clinical, and diagnostic aspects of the study. He is a highly respected investigator in the candidate's primary clinical area and will be very instrumental in providing access to Prechter study data and research support infrastructure. Dr. McInnis will also serve a key role in connecting the candidate to other important resources and opportunities at the depression center and beyond. He is a very experienced mentor.
- Dr. Murphy is a very established leader in her field and highly experienced mentor. She is ideally suited to serve as the primary mentor guiding training and development in the area of

micro-randomized trials and adaptive just-in-time interventions. She is a very experienced mentor.
- Dr. Kilbourne is a highly accomplished leader in the area of evidence-based interventions for people with serious mental illness and implementation of strategies that involve the use of phone-based outreach. She is a very experienced mentor.
- The candidate will gain concrete grant-writing experience working with Dr. McInnis and Kilbourne on an R01 application directly related to her field.

Weaknesses
- N/A

5. Environment and Institutional Commitment to the Candidate:
Strengths
- Outstanding institution with a proven record of producing top-notch junior investigators.
- Strong cross-departmental commitment to the candidate's growth, exposure to opportunities, and long-term success.
- Robust didactic and research infrastructure are made accessible to the candidate.

Weaknesses
- N/A.

Protections for Human Subjects:
Acceptable Risks and Adequate Protections
Data and Safety Monitoring Plan (Applicable for Clinical Trials Only):
- Acceptable

Inclusion of Women, Minorities and Children:
- Sex/Gender: Distribution justified scientifically
- Race/Ethnicity: Distribution justified scientifically
- For NIH-Defined Phase III trials, Plans for valid design and analysis: N/A
- Inclusion/Exclusion of Children under 18: Excluding ages <18; justified scientifically

Training in the Responsible Conduct of Research:
Acceptable

Comments on Format (Required):
Acceptable

Comments on Subject Matter (Required):
Acceptable

Comments on Faculty Participation (Required; not applicable for mid- and senior-career awards):
Acceptable

Comments on Duration (Required):
Acceptable

Comments on Frequency (Required):
Acceptable

Resource Sharing Plans:
- Unacceptable
- A complete resource sharing plan seems to be missing.

CRITIQUE 3:

Candidate: 2
Career Development Plan/Career Goals & Objectives: 3
Research Plan: 5
Mentor/Co-Mentor(s), Consultant(s), and Collaborator(s): 1
Environment and Institutional Commitment to the Candidate: 2

Overall Impact:
The candidate comes from a very strong mathematical background (PhD in mathematics, Cornell and 2 year post doc) and seeks to analyze the stochastic process of mood shifts in bipolar disorder to tailor psychosocial treatments in real time. The goal is to create a platform through which real time monitoring of mood and behavior uses mobile health tools, and use this to treat BP individuals in a timely manner. While this is not novel, the expertise of this junior researcher are; the researcher is currently at the intersection of mathematics and psychiatry, which is a unique position. It is clearly exciting that a strong mathematician has the interest and motivation to transition into psychiatry. Given the growing amount of data available in this high dimensional data age, it is essential for some people to have such hybrid knowledge, which this researcher is seeking.

She will first characterize evidence based metrics of mood course of individuals with BP and identify multidimensional metrics to identify patients at risk for “events.” Secondarily she will test the adherence and feasibility of using actigraphy on a mobile device to make real time treatment decisions in this patient population in a timely manner (in accordance with NIMH Strategy 2). Some details were vague but the overarching idea and end goal is exciting.

The candidate is brand new to the field of psychiatry and especially BP but seems highly committed. She has done research on using smart phone applications to monitor sleep habits, which is not dissimilar to the proposed research. Her training objectives are reasonable and her 2 co-mentors (renowned psychiatrist and statistician) are an excellent. Susan Murphy is a world renowned expert in psychiatric trial design and methodology development and would serve as an exemplary mentor for the proposed research.

While this is a very promising researcher at the intersection of mathematics and psychiatry, there was a question regarding a lack of ability to communicate difficult concepts to this audience. The concepts were often vague, e.g., it is not sufficient to state “mathematical modeling will be used to define markers.” Although the PI may understand the models she will use, she did not convey them clearly to the reader. Also of concern, and an area in which the PI will need training, is the lack of reference to prior statistical work in this subject area (only one statistical reference in the Bibliography). There appears to be a lack of working knowledge of biostatistics in general, and as it applies to modeling mood over time (e.g., sparse, irregular, and self report data) (e.g., via longitudinal data analysis, autoregressive models, time series analyses including Markov models, coupled and hierarchical hidden Markov models, as well as more advanced oscillatory methods she proposes that have been proposed in the literature). Finally, advanced courses in statistics could be useful as part of the training. These might include data mining, longitudinal data analysis, and advanced survival analysis courses, all of which she would be prepared to take given an incredibly strong background in probability theory.
The issues noted above are all addressable and enthusiasm for this application is high given the quality of mentorship, the potential impact of real time monitoring on BP treatment decision-making by a mathematician with strong probability theory background, and the devotion to transition into psychiatry.

1. Candidate:

Strengths

- Strong technical background including a PhD in Math from Cornell and a 2 year prestigious post doc in mathematics.
- Candidate has substantial expertise in the stochastic models she proposes to use to create an adaptive intervention.

Weaknesses

- Although she has applied similar mathematical models in other areas (including sleep and tissue injury), she has only been working in bipolar disorder for 1 year.
- Statistical treatments for this type of data are not referenced by the PI. It appears background and training in statistics; specifically statistical analysis of longitudinal mood data, are lacking.
- As the trainee is coming from a math background and has not worked on bipolar disorder previously, it is not surprising to be unfamiliar with the work already achieved in this area, but this could be remedied by more training.
- Vague statistical/technical terms are used throughout the application.

2. Career Development Plan/Career Goals & Objectives/Plan to Provide Mentoring:

Strengths

- Strong mentors and planned regular meetings with mentors are proposed.

Weaknesses

- Advanced statistics courses may be needed to fill gaps.

3. Research Plan:

Strengths

- Dimensional approach to modeling mood in BP seems important.
- Using real time metrics for mood and sleep (actigraphy) to make real time decisions is an exciting area of research.
- Adaptive interventions for mood disorders using real time data have been proposed for other diseases including BP, and harnessing smartphone technology via adaptive trials is a great opportunity to implement these for BP.
- The forthcoming work “Data-driven classification of bipolar I disorder from longitudinal mood data” is directly relevant to this application, and seems promising.
- The PI has extensive experience in the stochastic models proposed in Aim 1 (though little detail is given on them).
- Aims 2 and 3 will establish adherence and feasibility, respectively, of this monitoring system.

Weaknesses

- It will be difficult to communicate results of Aim 1 to the audiences who will benefit from them. The data analytics and models to be used were not clear from this application.
- The use of a micro randomized trial though it seems to make sense in this personalized/adaptive context might have benefited from more explanation.
- Aim 1 Study design: Electronic health record data will be used in Aim 1, but there is no mention of the biases and missing data that arise in this data type. EHR data can be difficult to assimilate and analyze to obtained unbiased inference.
- Aim 1 Study design: Only individuals who have completed assessments of mood longitudinally from EHR data will be included. With bipolar patients this may be as low as 30%, resulting in a selection bias. Similar criticism applies for the EHR outcome measures.
- For preliminary data, 178 BP patients were included from the Prechter Longitudinal Study of BD at the U-M. These had at least 24 self-reports. It is not stated how many patients were excluded from analysis. Again, there is concern of selection bias if only complete data are used (those completing all 24 longitudinal assessments may be in better health).
- Vague technical terms were used and it is unclear if PI can communicate findings to a non-technical clinical audience, or even semi-technical audience of applied statisticians.

4. Mentor(s), Co-Mentor(s), Consultant(s), Collaborator(s):
Strengths
- Co-Mentors and collaborators are renowned and have been working on adaptive implementation strategies for this population for 10-15 years.

Weaknesses
- None.

5. Environment and Institutional Commitment to the Candidate:
Strengths
- Environment and institutional commitment seem very good.

Weaknesses
- None.

Protections for Human Subjects:
Acceptable Risks and Adequate Protections
Data and Safety Monitoring Plan (Applicable for Clinical Trials Only):
- Acceptable

Inclusion of Women, Minorities and Children:
- Sex/Gender: Distribution justified scientifically
- Race/Ethnicity: Distribution justified scientifically
- For NIH-Defined Phase III trials, Plans for valid design and analysis: N/A
- Inclusion/Exclusion of Children under 18: Excluding ages <18; justified scientifically
- Clear breakdown of demographics given, all justified scientifically, exclusion of children, inclusion of women and minorities.

Training in the Responsible Conduct of Research:
Comments on Format (Required):
- Explicit plan for training including workshops and online training to be repeated every 2 years. Adherence to this will be important given the lack of human subjects background training this PI has had.

Comments on Subject Matter (Required):
Acceptable

Comments on Faculty Participation (Required; not applicable for mid- and senior-career awards):
Acceptable

Comments on Duration (Required):
Acceptable

Comments on Frequency (Required):
Acceptable

THE FOLLOWING SECTIONS WERE PREPARED BY THE SCIENTIFIC REVIEW OFFICER TO SUMMARIZE THE OUTCOME OF DISCUSSIONS OF THE REVIEW COMMITTEE, OR REVIEWERS' WRITTEN CRITIQUES, ON THE FOLLOWING ISSUES:

PROTECTION OF HUMAN SUBJECTS: ACCEPTABLE. Plans for the protection of human subjects are well considered.

INCLUSION OF WOMEN PLAN: ACCEPTABLE. Women will be well represented in the sample.

INCLUSION OF MINORITIES PLAN: ACCEPTABLE. Minorities will be well represented in the sample.

INCLUSION OF CHILDREN PLAN: ACCEPTABLE. Children under the age of 18 are appropriately excluded.

COMMITTEE BUDGET RECOMMENDATIONS: The budget was recommended as requested**.**

Footnotes for 1 K01 MH112876-01; PI Name: Cochran, Amy Louise

NIH has modified its policy regarding the receipt of resubmissions (amended applications). See Guide Notice NOT-OD-14-074 at http://grants.nih.gov/grants/guide/notice-files/NOT-OD-14-074.html. The impact/priority score is calculated after discussion of an application by averaging the overall scores (1-9) given by all voting reviewers on the committee and multiplying by 10. The criterion scores are submitted prior to the meeting by the individual reviewers assigned to an application, and are not discussed specifically at the review meeting or calculated into the overall impact score. Some applications also receive a percentile ranking. For details on the review process, see http://grants.nih.gov/grants/peer_review_process.htm#scoring.

MEETING ROSTER
National Institute of Mental Health Special Emphasis Panel

NATIONAL INSTITUTE OF MENTAL HEALTH
Interventions/Biomarkers
ZMH1 ERB-D (02)
09/09/2016

CHAIRPERSON(S)

SWARTZ, HOLLY A, MD
PROFESSOR
DEPRESSION & MANIC-DEPRESSION PREVENTION PROGRAM
WESTERN PSYCHIATRIC INSTITUTE AND CLINIC
PITTSBURGH, PA 15213

MEMBERS

BEN-ZEEV, DROR, PHD
ASSOCIATE PROFESSOR
DEPARTMENT OF PSYCHIATRY
DARTMOUTH PSYCHIATRIC RESEARCH CENTER
DARTMOUTH COLLEGE
HANOVER, NH 03755

DESANTIS, STACIA, PHD
ASSOCIATE PROFESSOR
DEPARTMENT OF BIOSTATISTICS
UT HEALTH SCIENCES CENTER AT HOUSTON
SCHOOL OF PUBLIC HEALTH
UNIVERSITY OF TEXAS
HOUSTON, TX 77030

DUFFECY, JENNIFER L, PHD
ASSOCIATE PROFESSOR
COLLEGE OF MEDICINE
DEPARTMENT OF PSYCHIATRY
UNIVERSITY OF ILLINOIS CHICAGO
CHICAGO, IL 60657

FRANK, MICHAEL J, PHD
ASSOCIATE PROFESSOR
DEPARTMENT OF COGNITIVE, LINGUISTIC AND PSYCHOLOGICAL SCIENCES
BROWN UNIVERSITY
PROVIDENCE, RI 02912

IOSIFESCU, DAN VLAD, MD
ASSOCIATE PROFESSOR
DEPARTMENT OF PSYCHIATRY
MOUNT SINAI SCHOOL OF MEDICINE
NEW YORK, NY 10029

SCHMIDT, NORMAN BRAD, PHD
PROFESSOR
DEPARTMENT OF PSYCHOLOGY
FLORIDA STATE UNIVERSITY
TALLAHASSEE, FL 32306

SHIN, LISA MARIE, PHD
PROFESSOR AND DEPARTMENT CHAIR
DEPARTMENT OF PSYCHOLOGY
TUFTS UNIVERSITY
MEDFORD, MA 02155

SLOAN, DENISE M., PHD
PROFESSOR
DEPARTMENT OF PSYCHIATRY
SCHOOL OF MEDICINE
BOSTON UNIVERSITY
BOSTON, MA 02130

SOUTHAM-GEROW, MICHAEL A, PHD
PROFESSOR OF PSYCHOLOGY AND PEDIATRICS
DEPARTMENT OF PSYCHOLOGY
VIRGINIA COMMONWEALTH UNIVERSITY
RICHMOND, VA 23284

THOMPSON, WESLEY K, PHD
ASSOCIATE PROFESSOR IN RESIDENCE
DEPARTMENT OF PSYCHIATRY
UNIVERSITY OF CALIFORNIA, SAN DIEGO
LA JOLLA, CA 92093

SCIENTIFIC REVIEW OFFICER

SOMMERS, DAVID I., PHD
SCIENTIFIC REVIEW OFFICER
DIVISION OF EXTRAMURAL ACTIVITIES
NATIONAL INSTITUTE OF MENTAL HEALTH
NATIONAL INSTITUTES OF HEALTH
ROCKVILLE, MD 20852-9606

EXTRAMURAL SUPPORT ASSISTANT

MUNDELL, TERRY
EXTRAMURAL SUPPORT ASSISTANT
DIVISION OF EXTRAMURAL ACTIVITIES
NATIONAL INSTITUTE OF MENTAL HEALTH
NATIONAL INSTITUTES OF HEALTH
BETHESDA, MD 20892

Consultants are required to absent themselves from the room during the review of any application if their presence would constitute or appear to constitute a conflict of interest.
